# Mismatches in youth sports talent development

**DOI:** 10.3389/fspor.2023.1189355

**Published:** 2023-06-16

**Authors:** Humberto M. Carvalho, Carlos E. Gonçalves

**Affiliations:** School of Sports, Federal University of Santa Catarina, Florianópolis, Santa Catarina, Brazil

**Keywords:** growth, maturation, relative age effect, selection, youth sports

## Introduction

The growing pressure to identify and nurture talented athletes for adult competitions has led sports organizations to invest significant efforts in identifying markers of talent at increasingly younger ages (1, 2). Initially, coaches were responsible for this task, but over time, it has shifted towards sport scientists (3, 4). However, in most cases, the final decision regarding the evaluation and development of young prospects still rests with the coaches (5).

A significant challenge in the pursuit of a reliable predictive model for adult performance is the emergence of the “biologic genotype,” which suggests that genetics plays a partial role in the physical, physiological, or anthropometric traits necessary for athletic success (6). This phenomenon occurs during childhood and adolescence, coinciding with the period of sport specialization (6, 7). Alongside contextual factors, three major interrelated developmental problems arise when considering a viable model of talent identification and development: growth and maturation, relative age effect, and maturation and training loads. These problems have gained increased awareness in the context of youth sports and talent identification and development (8–10). In particular, there has been a recent discussion emphasizing the importance of maturation and relative age in talent development (11). Here, we extend the discussion to address often overlooked assumptions and their potential implications for researchers and coaches’ interpretations.

## Growth and maturation

The first process is the phenotypic process of pubertal changes, namely growth and maturation. The significant influence of growth and maturation on body size, physical function and performance, psychological, social, and behavioral characteristics has been widely recognized (8, 12, 13). Young athletes are often considered to have relatively homogeneous maturity status, training experience, body dimensions, functional capacity, and sport-specific skills (13). When a combination of size, strength, power, and endurance are determining factors in sports such as basketball or football, there tends to be an over-representation of early-maturing players (14, 15). On the other hand, in sports where smaller body size and relative strength are determining factors, such as gymnastics, or in sports where late specialization and stature are common, such as volleyball, late-maturing players are more represented (16, 17). The interpretation of the growth and maturation of young athletes is crucial in the selection process, especially in talent development contexts. For example, it has been noted the potential pitfalls of maturity-associated bias on youth selection (15). Nevertheless, there is limited retrospective data in talent development contexts with skeletal maturity assessments. Likely, open-science practices and data sharing (18) will help to improve the strength of evidence in youth sports research, particularly in talent development contexts.

Longitudinal data monitoring of young athletes’ growth and development is scarce, and mostly limited to stature and body mass (19). Interpretations of the occurrence of biological milestones such as peak height velocity or age at menarche require longitudinal observations and advanced modeling techniques. There are several practical problems with longitudinal studies, and even more challenging in applied youth sports settings (20). Recently, several advances have been made in fitting complex longitudinal data, including dealing with imbalanced data, and increased awareness of the strengths, assumptions, and limitations of different modeling approaches (19, 21). These advances have been made possible by increased computational resources, allowing for recent discussions on modeling methods comparisons (21–23).

However, interpretations of the variation in size, performance, and behavior of young athletes associated with growth and maturity are mostly based on cross-sectional data. Prediction-based equations, such as the maturity offset equations (24, 25) or percentage of mature (adult) stature without using skeletal age (26, 27), provide an alternative to having a reference of maturity status when considering cross-sectional observations. These methods are non-invasive and easy to measure. However, the risk of measurement error of anthropometric measures can be a concern in applied settings. On the other hand, these methods were derived from specific populations, mostly North American Caucasians (24–27). Hence, there is limited validity for the use of prediction-based equations in applied youth sports settings, and even more in talent development research. The limitations of prediction-based equations have been discussed (28, 29), also considering contexts of youth sports (30). However, researchers often overlook that these methods are potentially insensitive, and a young athlete may have been assigned to the wrong maturity status category (11, 12).

“Quick fixes” to interpret maturity status and timing based on non-invasive estimates are limited (31), despite their generalized interest and use in youth sports research and applied contexts. Therefore, it is important to exercise care in study designs and measurements, recognize and incorporate method assumptions and limitations, and keep interpretations conservative. Further and deeper development and validation of non-invasive indicators of maturity status and timing remain key issues in youth sports research. In particular, hierarchical/multilevel modeling using a fully Bayesian framework (32, 33) offers a robust and flexible approach to combine available longitudinal data from youth sport-specific samples with well-known shapes and variation in pubertal growth from available growth data (contemporary or otherwise) (19).

Recently, the application of bio-banding in the talent development context of youth sports has been advocated and applied in professional clubs or academies in the search for young “elite” athletes (9). The approach involves grouping and/or evaluating athletes based on their maturity status (and/or body size) rather than chronological age (9). Data-driven interpretations of bio-banding application are becoming more frequent in talent development contexts (particularly in youth football) [e.g., (34–36)]. At face value, the validity of the approach may seem reasonable. However, its application in research and real-world contexts relies on estimated maturity status based on prediction-based equations. Therefore, it is crucial to gather sufficient data on the application of bio-banding in youth sports and examine the accuracy of maturity status estimations in order to engage in meaningful discussions about its validity.

## Relative age effect

The second bias is the phenomenon of Relative Age Effect (RAE). Albeit being the object of an extensive body of literature (37–40), RAE persists as coaches continue to be lured by apparent advantages of relative older athletes. RAE bias can appear as early as around 6 years of age in youth football (40). Starting from the onset of sport participation in childhood to early adolescence, around 14–15 years, coaches can engage in a chain of decisions to select or deselect participants based on their date of birth. From a talent development perspective, the exclusion of potential talents or the inclusion of future non achievers represent a negative side effect of a chronologically-based decision.

Unlike maturity status, RAE is easy to assess and offers a field for quantitative studies about the persistence of the phenomenon in adult sport. The observation of the RAE bias in the top levels of competition is highly dependent of the type of sport [e.g., (37–39)]. However, the general trend points to the disappearance of the effect at adult high-level of performance (37).

Our own research (7) revealed that being born in the first quarter of the year did not have an influence on athletic performance. Even when an initial advantage was observed, it diminished rapidly. By late adolescence, typically around 17 years of age, the best scores in any performance test were unrelated to the quarter of birth. These findings provide further evidence that the RAE and maturity status should not be confounded. However, the observations suggest a phenomenological emergence of the “survival of the fittest” (41). As at least for boys, the older individuals, both chronologically and biologically, appear to be more likely to be retained by coaches.

The outcomes are more a consequence of the athletes’ responses to the training loads and to the ecologies of practice than determined by a particular characteristic like the birth quarter, maturity status or the year of engagement in talent development programs. Hence, the challenge remains to limit the potential bias associated to RAE on young athletes selection/exclusion, particularly at early ages.

## Maturation and training loads

The third bias is represented by the interaction between maturation and the training load. We focus on two issues: influence of training exposure on developmental changes in performance, and monitoring training loads and maturity status.

Coaches and researchers know that metabolic capacities are altered and enhanced by continued training through biological adaptations. When measuring of developmental changes during the specialization years, the maturation process acts as a confounding factor when interpreting eventual improvements in performance associated to training exposure (42). Furthermore, chronological age, biological age and sport age (accumulated training experience) interact and influence performance development with varying patterns across time (43, 44). It is well known that aerobic capacity, translated in the development of the endurance capabilities, increases through childhood and adolescence (8). The same is true for short-term muscle power outputs, observed and measured as strength or speed. Short-term muscle power outputs increases at the onset of puberty, as the growth of muscle mass is strongly dependent of the maturation process (45). However, data tracking developmental changes in young athletes adjusting for growth, maturation and training exposure is scarce, and merits further study (42), particularly in talent development context.

On the other hand, researchers are well aware of the obstacles raised by the multidimensional nature of performance and by the demands of each specific sport (2, 4, 46). Nevertheless, the pursuit of predicting models to identify those athletes more likely to succeed in adult sport remain a key interest of youth sport researchers (47–49). Multiple sets of tests were designed to measure biological characteristics, and/or functional characteristics at various age groups. However, the results in physical tests are strongly dependent of the accumulated hours of training, and of the respective training load (besides the fact that the assessment is often made downstream of the moment of selection). For instance strength development is connected both to the maturation process of testosterone production and to the participation in organized training sessions. Furthermore, there are different paces in maturation for boys and girls (8).

There is a large body of data describing training loads monitoring in talent development environments, particularly in youth football (50). Recently, the influence of maturation on training loads responses of young athletes in talent development contexts has draw attention [e.g., (51, 52)]. Exposition to high and demanding training loads raises concerns associated to injury risk, particularly during the periods of accelerated pubertal growth (52). As noted earlier, the use of non-invasive predictive equations hinder the potential interpretations. Unfortunately, this has been the case in most of the available research focusing on the relations between maturation and training loads among young athletes in talent development contexts [e.g., (53, 54)].

## Future directions

To allow meaningful interpretations of young talented athletes data, we focus our suggestion to researches on three key issues:

(i) Adopting open-science and data sharing practices, allowing to overcome the expected small samples sizes reported, and combination of different sources of information;
(ii) Go beyond statements about the limits of non-invasive predictive methods of somatic maturity status, and explore advanced modeling approaches to include information and critically assess the models and inferences;
(iii) Consider theoretical lenses to frame questions, models and interpretations of potential mismatches between young athletes, and within-athlete development.

The potential biases associated to growth, maturation, RAE and training loads are especially challenging for coaches, who must evaluate their athletes’ performances on a daily basis. Furthermore, the decisions made by coaches, as perceived by young athletes, are not limited to selection or exclusion but also involve micro-management of training sessions and competitions (such as playing time, praise and critique, composition of groups, promotion to higher levels, etc). On the other hand, the structures of talent development settings vary in terms of their human resources, sport types, and overall organization. In professional sports, talent development facilities have the capacity to recruit, support, and prepare the best prospects, and professional coaches are likely to benefit from the counseling of a performance analysis team. Even in such situations, the traps of maturity status and RAE are still present and can lead to decisions made without scientific or logical basis.
